# Sexual dysfunctions in patients with well-compensated chronic liver disease: role of etiology, Mediterranean diet and quality of life in an observational cross-sectional study

**DOI:** 10.1093/sexmed/qfaf025

**Published:** 2025-04-24

**Authors:** Lorenzo Romano, Mariano Fonticelli, Filomena Morisco, Kateryna Priadko, Alba Rocco, Gerardo Nardone, Luisa Ranieri, Luigi Napolitano, Felice Crocetto, Biagio Barone, Davide Arcaniolo, Lorenzo Spirito, Celeste Manfredi, Antonietta Gerarda Gravina, Carmine Sciorio, Antonio Tufano, Antonio Cioffi, Ferdinando Fusco, Marco Romano, Marco De Sio

**Affiliations:** Department of Woman, Child and General and Specialized Surgery and Urology Unit, University of Campania “Luigi Vanvitelli”, Naples, 80138, Italy; Urology Unit, Ospedale del Mare, Naples, 80147, Italy; Department of Precision Medicine and Hepatogastroenterology Unit, University of Campania “Luigi Vanvitelli”, Naples, 80138, Italy; Gastroenterology Unit, Department of Clinical Medicine and Surgery, Federico II University, Naples, 80131, Italy; Department of Precision Medicine and Hepatogastroenterology Unit, University of Campania “Luigi Vanvitelli”, Naples, 80138, Italy; Gastroenterology Unit, Department of Clinical Medicine and Surgery, Federico II University, Naples, 80131, Italy; Gastroenterology Unit, Department of Clinical Medicine and Surgery, Federico II University, Naples, 80131, Italy; Gastroenterology Unit, Department of Clinical Medicine and Surgery, Federico II University, Naples, 80131, Italy; Department of Neurosciences, Reproductive Sciences and Odontostomatology and Urology Unit, Federico II University, Naples, 80131, Italy; Department of Neurosciences, Reproductive Sciences and Odontostomatology and Urology Unit, Federico II University, Naples, 80131, Italy; Department of Neurosciences, Reproductive Sciences and Odontostomatology and Urology Unit, Federico II University, Naples, 80131, Italy; Department of Woman, Child and General and Specialized Surgery and Urology Unit, University of Campania “Luigi Vanvitelli”, Naples, 80138, Italy; Department of Woman, Child and General and Specialized Surgery and Urology Unit, University of Campania “Luigi Vanvitelli”, Naples, 80138, Italy; Department of Woman, Child and General and Specialized Surgery and Urology Unit, University of Campania “Luigi Vanvitelli”, Naples, 80138, Italy; Department of Precision Medicine and Hepatogastroenterology Unit, University of Campania “Luigi Vanvitelli”, Naples, 80138, Italy; Urology Unit, Alessandro Manzoni General Hospital, Lecco, 23900, Italy; Urology Unit, Istituto Nazionale Tumori, IRCCS Fondazione G Pascale, Naples, 80131, Italy; Urology Unit, Ospedale del Mare, Naples, 80147, Italy; Urology Unit, A.O. Sant'Anna e San Sebastiano, Caserta, 81100, Italy; Department of Precision Medicine and Hepatogastroenterology Unit, University of Campania “Luigi Vanvitelli”, Naples, 80138, Italy; Department of Woman, Child and General and Specialized Surgery and Urology Unit, University of Campania “Luigi Vanvitelli”, Naples, 80138, Italy

**Keywords:** sexual dysfunctions, Mediterranean diet, quality of life, chronic liver disease, MASLD-FSFI, Iief

## Abstract

**Background:**

Sexual dysfunctions (SD) are highly prevalent in Chronic Liver Diseases (CLD). Whether Metabolic dysfunction-Associated Steatotic Liver Disease (MASLD) carries a higher risk of SD is unknown as is the role of dietary patterns or quality of Life (QoL).

**Aim:**

to assess (1) prevalence of SD in CLD; (2) whether MASLD is a risk factor for SD; (3) the role of adherence to Mediterranean Diet (MD) or QoL.

**Methods:**

Observational, cross-sectional study, 207 CLD patients (84 females and 123 males), median age 57 years (IQR:46-63); 96 (46.4%) MASLD; and 111 (53.6%) nonMASLD.

**Outcomes:**

SD were assessed through Female Sexual Function Index (FSFI) and International Index of Erectile Function (IIEF) questionnaires. Adherence to MD was evaluated by the MD Score, QoL by SFHS-12 questionnaire evaluating physical [(ie, Physical Component Summary (PCS)] and mental [(ie, Mental Component Summary (MCS)] health. Multivariate analysis identified predictors of SD.

**Results:**

(1) SD prevalence in CLD was 157/207 (75.8%); 80.9% females were at risk for SD, altered sexual desire/arousal and dyspareunia being the most common complaints, whereas 72.3% males had erectile dysfunction (ED); (2) prevalence of SD was higher in MASLD (89%) than in nonMASLD (64%) (*P* < 0.001); (3) in females, at univariate analysis, a negative correlation was found between FSFI and age, hypertension, or MASLD; (4) in males, at univariate analysis, IIEF-ED negatively correlated with age, DM2, or MASLD, whereas positively correlated with PCS and MCS; (5) in females, at multivariate analysis BMI (OR = 0.779,CI 95% = 0.640-0.948) and MCS (OR = 0.840,CI 95% = 0.741-0.953) were protective against SD, whereas age (OR = 1.115,CI 95% = 1.040–1.263) and DM2 (OR = 120.894,CI 95% = 1.396–10 741) were predictive of SD; (6) in males, at multivariate analysis, age (OR = 1088,CI 95% = 1032-1.148) and MASLD (OR = 4.075,CI 95% = 1.120-14.828) were risk factors for, whereas PCS (OR = 0,928,CI 95% = 0,865-0,995), and disease duration (OR = 0.393,CI 95% = 0.187-0.822) were protective against SD; 7) MD adherence, while higher in nonMASLD vs MASLD (*P =* 0.004), was not an independent protective factor against SD.

**Clinical Implications:**

SD should not be underestimated in CLD patients, in particular those with MASLD.

**Strengths and Limitations:**

Comprehensive study evaluating SD in a large cohort of CLD patients of both sexes, comparing MASLD vs nonMASLD. Due to its cross-sectional design, no conclusions can be drawn about cause and effect.

**Conclusions:**

(1) CLD, in particular MASLD, have a high prevalence of SD which is not affected by MD adherence, whereas QoL seems to play a role; (2) CLD patients should be evaluated for SD, for early diagnosis and treatment.

## Introduction

Sexual dysfunctions (SD) may have a relevant impact at the physical and emotional well-being level both in females and males. In the general population, almost 40% to 50% of women, irrespective of age, experience at least one SD ^1^. On the other hand, prevalence of SD in men, in particular erectile dysfunction (ED) largely depends on age, ranging from 1% to 10% in those younger than 40 years and reaching up to 50% -100% in those of more than 70 years of age.^1^ An international survey including 13 882 women and 13 618 men of 40 to 80 years of age, showed that altered sexual function is closely related to physical health.^2^ SD, when associated with chronic comorbidities in particular those with a global increasing prevalence, lead to a significant decrease in quality of life (QoL) and require a multidisciplinary approach.^3^

Female sexual dysfunctions (FSD) include sexual desire, arousal problems, inadequate lubrication, and pain during sex, all of which have a negative impact on QoL, mental health, and relationships with sexual partners.^4^ In men, ED (i.e., the inability to achieve or maintain an erection suitable for satisfactory sex) represents the most important alteration of male sexual function and may recognize different pathophysiologic mechanisms, whose incidence and prevalence is strongly related to increasing age and presence of comorbidities.^4^^,^^5^ In both sexes, among other factors, unhealthy lifestyle habits, including diet, physical inactivity, smoking and alcohol abuse may play a significant role in the development of SD.^6^^,^^7^ Diet is emerging as an important determinant in several pathological conditions and Mediterranean diet (MD) has been shown to be a healthy dietary pattern associated with an improvement of sexual function in both males and females.^8–10^

Chronic hepato-gastroenterological disorders have been described to be associated with an increased prevalence of SD.^11^ In particular, an altered sexual function has been reported in patients with celiac disease or inflammatory bowel disease.^12^^,^^13^

Chronic hepatitis (CH) refers to a number of liver disorders with a different etiology (ie, viral, autoimmune, altered metabolism, alcohol abuse, drugs, metabolic, iron or copper overload) and different severity, in which inflammation and necrosis of liver cells continue for long time. The advanced stage of CH is liver cirrhosis, a condition characterized by extensive liver fibrosis, formation of hepatocyte nodules surrounded by connective tissue and different degree of liver function impairment. Metabolic dysfunction-Associated Steatotic Liver Disease (MASLD), previously referred to as Non-Alcoholic Fatty Liver Disease (NAFLD), represent the most prevalent liver disorder in the Western countries, being one of the leading causes of orthotopic liver transplantation (OLT).^14^ This condition, primarily affects subjects with obesity or type 2 diabetes mellitus (DM2), now considered a component of the Metabolic Syndrome (MetS) which, in turn, is a risk factor for SD.^15^^,^^16^ Several studies have shown that patients with chronic liver diseases (CLD) are at higher risk of SD than the general population, with ED and decreased sexual desire in men, altered sexual desire and arousal, and dyspareunia in women, being the most commonly reported alterations of sexual function.^16–25^ While the prevalence of SD in CLD has clearly been shown to mainly depend on the degree of liver function impairment,^17–20^ the role of etiology is still debated.^11^ In particular whether MASLD patients carry a higher risk of SD compared to non MASLD patients has never been evaluated systematically in a large cohort of patients.

Moreover, adherence to MD is associated to a lower prevalence of MetS.^26^ Whether adherence to MD plays a protective role against SD in CLD patients is not completely clear. Finally, the role of QoL as a predictor of SD in patients with CLD of different aetiology is not fully understood.

Our working hypothesis is the following: are SD highly prevalent in CLD and does MASLD play a relevant role in this clinical setting? Also, what is the influence of dietary patterns and QoL? This might be relevant as to a more comprehensive management of patients suffering from CLD. Therefore, this multicenter, observational, cross-sectional study was designed in order to assess the prevalence of SD in CLD patients. As a secondary end-point we sought to evaluate whether the prevalence of SD was different between patients with MASLD or non MASLD origin. Finally, the role of a number of clinical-demographic variables as well as of adherence to MD, or QoL as predictors of normal or altered sexual function in this clinical setting was investigated.

## Materials and methods

### Study design and population

This is a multicenter cross-sectional observational study involving patients attending the Gastroenterology and Liver Units at University of Campania “Luigi Vanvitelli”, Naples, and University Federico II, Naples, Italy.

To be eligible for the enrollment in the study, participants had to be adults (18 years or older) with well established, well-compensated CLD.^27^^,^^28^ Well compensated CLD was defined according to the Child-Pugh criteria^27^^,^^28^ and all of the enrolled patients were in Child-Pugh Class A 5 score. We excluded from the study: legal unable patients to provide free consent for attendance, patients with diagnosed psychiatric disorders or already known sexual disorders. The following clinical-demographic variables were collected through an on-line questionnaire administered at the same time of those exploring MD, QoL and SD: age, gender, level of education, type of work, total number of partners, body mass index (BMI), smoking habits, consumption of alcohol, diagnosis of diabetes and/or, hypertension, type of CLD [(ie, MASLD, viral, alcoholic, autoimmune, haemochromatosis, Wilson disease), stage (ie, presence and degree of fibrosis through Fibroscan evaluation^29^), duration of the disease, and ongoing therapy. All participants completed the study questionnaires anonymously, considering sex stratification into males and females.

This study was conducted according to the principles of the World Medical Association Declaration of Helsinki. Informed consent was obtained from all individual participants included in the study. Furthermore, the study was approved by our Departmental Ethics Committee.

### Assessment of sexual dysfunction in the female population

Female Sexual Function Index (FSFI) questionnaire,^30^ was used to assess SD in women. This questionnaire consists of 19 questions, each of which is part of a domain exploring: sexual desire (questions 1 and 2, scores 1-5 and multiplication factor 0.6), sexual arousal (questions 3–6, scores 0-5 and multiplication factor 0.3), lubrication (questions 7–10, scores 0-5 and multiplication factor 0.3), orgasm (questions 11–13, scores 0–5 and multiplication factor 0.4), sexual satisfaction (questions 14–16, scores 0–5 and multiplication factor 0.4), and pain associated with sexual activity (questions 17-19, scores 0-5 and multiplication factor 0.4). Each domain has a minimum score (0 or 1.2) up to a maximum score of 6. Each domain’s score is multiplied by a correction coefficient, resulting in a domain subscore, which are all added to obtain the final score (from a minimum of 2 to a maximum score of 36). The cut-off limit considered to indicate women at risk for FSD was ≤26.5.^30^

### Assessment of sexual dysfunction in the male population

Male patients completed the 15-item International Index of Erectile Function (IIEF-15), a validated, multidimensional, self-administered questionnaire that investigates male sexual function in the last 4 weeks.^31^ To each of the 15 questions is assigned a score from 0 to 5; they examine the main domains of male sexual function: erectile function (questions 1–5 and 15, total scores 1–30), orgasmic function (questions 9 and 10, total score 0–10), sexual desire (questions 11 and 12, total scores 2–10), relationship satisfaction (questions 6–8, total score 0-15) and overall satisfaction (questions 13 and 14, total scores 2–10). Based on the results of the erectile function IIEF (questions 1–5 and 15) we can classify ED as severe (first domain scores: 6–10), moderate (11–16), mild (17–25) and absent (26–30). To define whether other aspects of male sexual function were compromised, we arbitrarily considered, as a cut-off value, a score corresponding to less than 83% of the total score for each of the remaining domains explored by IEEF-15 (ie, comparable to the percent cut-off value used to define ED).^13^ This is an arbitrary score and is not a cut-off value validated enough to establish diagnosis or severity of a specific SD other than ED.

### Assessment of adherence to MD

The degree of adherence to the MD was assessed by the 9-point scale by Trichopoulou et al.^32^ Each domain is assigned a value of 0 or 1 as follows: about beneficial components (vegetables, legumes, fruit and nuts, cereals and fish) people who had a consumption below average received a score equal to 0, whereas people whose consumption was equal to or higher than the average received a score of 1. As for the presumed harmful components (meat, poultry and dairy products) patients who had a consumption lower than average, received a score equal to 1; people whose consumption was equal to or above average received a score of 0. For ethanol, a score of 1 was assigned to males who consumed between 10 and 50 grams per day and to females who consumed between 5 and 25 grams per day. With regard to fat intake, we used the ratio between monounsaturated lipids and saturated lipids. Therefore, the total score of the MD ranged from 0 to 9: patients with a score > 6 were stratified as subjects with “maximum adherence to the MD”, while those with a score *<* 6 as subjects with “minimal adherence to the MD”.

### Assessment of quality of life

QoL of participants in the study was evaluated by using the SF-12 (Short Form Health Survey), a validated scale that allows to measure the physical and mental health status of respondents.^33^ SF-12 consists of a series of 12 Likert scale questions, each of which is designed to measure a specific aspect of physical health (physical activity, role limitations due to physical health, physical pain, general health), and mental health (vitality, social activities, emotional state, mental state, general health). Through appropriate statistical analysis techniques,^34^ the information obtained from the individual questions is traced back to two synthetic numerical indices: the state of physical (PCS) and mental (MCS) health of people. The international reference values for these two synthetic indices are 50 + 10 (Mean + Standard Deviation).^33^

### Sample size and statistical analysis

For sample size calculation we considered the estimated prevalence of altered FSFI and IEEF in the general population of comparable age. Assuming a prevalence of FSFI of 40%–50% and a prevalence of ED of 40%–60% in the general population, an expected prevalence in the study of approximately 70% with a statistical power of 80% and a 2-sided significance level of 0.05, the minimum number of subjects for an adequate study corresponded to 167 patients. The sample size was increased to 207 to account for drop-out of up to 20% and to give more power to the study.

Descriptive statistics were used to present the data. The continuous variables were expressed as median and the interquartile (ie, Q1 and Q3) range, while the discrete variables (ordinal and categorical) were expressed as number and percentage. The distribution of the variables was tested using the Kolmogorov–Smirnov test to assess their normality and to choose between parametric and non-parametric tests. Categorical variables were compared using the Chi-square test, if ≤20% of expected cell counts are less than 5 or the number of samples is at least 100, or Fisher’s exact test if >20% of expected cell counts are less than 5 and the number of samples is less than 100, while the continuous and ordinal variables (discrete but sortable quantitative variables) were differentiated between groups (qualitative variables) and subgroups using the Mann–Whitney U test or the Kruskal-Wallis H test, depending on the degrees of freedom of the variable related to the group/subgroup. The strength of the correlation between two quantitative variables was examined using the Spearman Correlation rho test. Predictive factors of SD were identified by multivariate linear/logistic regression analysis. Dependent variables (i.e.FSFI-19 and IIEF-15 total scores) were compared with independent variables (ie, age, smoke, alcohol, DM2, BMI, hypertension, MASLD, nonMASLD, fibroscan score, duration of CLD, MD, PCS, and MCS and results are illustrated as odds ratio (OR) with 95% confidence interval (CI). A p-value of <0.05 was considered statistically significant. Data were recorded and statistically analyzed using the Statistical Package for Social Science software (IBM SPSS® Version 26.0.1.0 software - IBM Corp., Armonk, NY, USA).

## Results

### Clinical-demographic characteristics

We enrolled 207 patients, 123 (59.4%) men and 84 (40.6%) women. Median age was 57 (46–63) years, with a median BMI value of 26.2 (23.5-29.2) kg/m^2^; 119 patients (57.5%) never smoked, whereas 174 (84%) did not consume alcohol. Among comorbidities, 39 patients (18.8%) had DM2, and 87 (42%) had arterial hypertension.

Regarding the type of CLD, 111 patients (53.6%) had nonMASLD-aetiology (45 HBV, 38 HCV, 9 hemochromatosis and 19 autoimmune) and 96 (46.4%) had MASLD. The median duration of CLD was 4 (1–10) years. MASLD group consisted of 61 males and 35 females, whereas the nonMASLD group of 62 males and 49 females. Prevalence of SD in our cohort of CLD patients was in total 157/207 (75.8%). In detail, prevalence of SD was 71/111 (64%) in patients with nonMASLD aetiology and 86/96 (89%) in patients with MASLD (*P* < 0.001).

As for the adherence to MD, the global median score was 4 (3–5), with 37 patients (17,9%) with a maximal adherence (ie, with a global score of at least 7/9).

Median score for PCS and MCS was 44.9 (38.6-53.6) and 42.8 (35.9-50.0), respectively. This is below the median value obtained in a cohort of healthy controls 50 (40–60).^35^

A summary of clinical-demographic characteristics stratified by gender and type of CLD, is illustrated in Table 1 and Table 2, respectively. In particular, prevalence of smoking and alcohol consumption were significantly higher in the male population compared with females (48.8% vs 33.3%, *P =* 0.027 and 24.4% vs 3.6%, *P* < 0.001, respectively) (Table 1). BMI values were significantly higher in MASLD patients compared to nonMASLD ones, [27.3 (25.3-29.5) vs 25.0 (23.1-27.7) *P* < 0.001, respectively]. A significantly higher percentage of MASLD- patients had DM2 (27.1% vs 11.7%, *P =* 0.005) and lower MD scores compared to nonMASLD patients [3.0 (2-4) vs 4.0 (3-5), *P =* 0.004, respectively]. Finally, MCS score was slightly but significantly higher in nonMASLD group compared to those with MASLD [45.1 (37.6-51.3) vs 40.0(33.2-47.6), *P =* 0.001, respectively].

**Table 1:** Clinical-demographic characteristics of our cohort of 207 Chronic Liver Disease (CLD) patients stratified by gender.

	**Females** (N = 84) Median (IQR) or n (%)	**Males**(N = 123) Median (IQR) or n (%)	**p-value**
Age (yrs)	55 (45-62)	57 (48.5-65)	0.335
BMI	26.3 (23.3-29.4)	26.3 (23.7-28.7)	0.575
Smoke	28 (33,3%)	60 (48.8%)	**0.027**
Alcohol	3 (3.6%)	30 (24%)	**<0.001**
DM2	15 (17.9%)	24 (19.5%)	0.765
Hypertension	37 (44%)	50 (40.7%)	0.627
CLD			
nonMASLD MASLD	49 (58.3%) 35 (41.7%)	62 (50.4%) 61 (49.6%)	0.261
Fibroscan (kPa)	9.9 (5-12.8)	10.2 (6-13.6)	0.706
MD score	4 (3-5)	4 (2-5)	0.091
PCS score	44.6 (38.6-51.3)	45.4 (38.8-54.5)	0.318
MCS score	41.2 (35.8-48)	45.1 (35.9-50.2)	0.318

IQR: Inter Quartile Range; BMI: Body Mass Index; DM2: type 2 Diabetes Mellitus; CLD: Chronic Liver Disease; MASLD: Metabolic dysfunction-Associated Steatotic Liver Disease; kPa: kiloPascal; MD: Mediterranean Diet; PCS: Physical Component Summary; MCS: Mental Component Summary

*P* < 0.05 is considered statistically significant

**Table 2:** Clinical-demographic characteristics of our cohort of 207 Chronic Liver Disease (CLD) patients stratified by type of CLD.

	**nonMASLD** (N = 111) Median (IQR) or n (%)	**MASLD** (N = 96) Median (IQR) or n (%)	**p-value**
Age	59 (46.5-64)	54.5 (45.8-62)	0.241
BMI	25 (23.1-27.7)	27.3 (25.3-29.5)	**<0.001**
Smoke	46 (41.4%)	42 (43.8%)	0.738
Alcohol	12 (10.8%)	21 (21.9%)	**0.030**
DM2	13 (11.7%)	26 (27.1%)	**0.005**
Hypertension	42 (37.8%)	45 (46.9%)	0.189
Gender			
Male Female	62 (55.9%) 49 (44.1%)	61 (63.5%) 35 (36.5%)	0.261
Fibroscan (kPa)	12.1 (6-44)	13.2 (8.1-38)	0.989
MD score	4 (3-5)	3.0 (2-4)	**0.004**
PCS score	47.2 (38.6-54.3)	43.1 (38.9-51.3)	0.132
MCS score	45.1 (37.6-51.3)	40 (33.2-47.6)	**0.001**

MASLD: Metabolic dysfunction-Associated Steatotic Liver Disease; IQR: Inter Quartile Range; BMI: Body Mass Index; DM2: type 2 Diabetes Mellitus; kPa: kiloPascal; MD: Mediterranean Diet; PCS: Physical Component Summary; MCS: Mental Component Summary

*P* < 0.05 is considered statistically significant

### Sexual function in the female population

FSFI median score in our female CLD population was 19.65 (8.85-25.1). By adopting the cut-off value of *<*26,5 to identify females at risk of SD, prevalence of FSD risk was 68/84 (80.9%). Specifically, by analyzing the single domains of the FSFI, we identified as areas of greatest sexual concern to the majority of participants, sexual desire in 79/84 (94%), arousal in 69/84 (82.1%), lubrication in 62/84 (73.8%), orgasm in 67/84 (79.7%), intercourse satisfaction in 58/84 (69%), and sexual pain in 68/84 (80.9%) patients (data not shown). Table 3 shows FSFI score differences between subgroups. In particular hypertension and a low degree of education were significantly associated to lower FSFI scores. Also, a significantly lower FSFI total score was observed in MASLD than in nonMASLD patients (Table 3).

**Table 3:** Female Sexual Function Index (FSFI) scores and clinical-demographic variables.

**Variables**	**N and Frequency (%)**	**FSFI Total score (IQR)**	**p-value**
Smoke	28 (33.3%)	21.25 (14.4-24.3)	0.563
Alcohol	3 (3.6%)	14.8 (8.4-19.5)	0.417
CLD nonMASLD MASLD	49 (58.3%) 35 (41.7%)	21.8 (10.5-27.3) 16.8 (6-21.8)	**0.023**
Fibroscan kPa <7.2 k 7.3-12.5 12.6-17.5 >17.5	24 (28.6%) 18 (21.4%) 13 (15.5%) 29 (34.5%)	21.6 (14.9-27.8) 18.8 (7.5-23.5) 18.3 (13.9-24.1) 20.4 (8.1-24.2)	0.587
			
DM2	15 (17.9%)	18.3 (11.5– 20.4)	0.280
Hypertension	37 (44%)	18.3 (6.5-22.4)	**0.042**
			
Duration of Disease < 5 years 5-10 years > 10 years	42 (50%) 23 (27.4%) 19 (22.6%)	19.5 (8.4 –24.2) 19 (7.7-23,8) 22.4 (14.3-26.2)	0.929
Education Unschooled Primary School Secondary School High School Degree	1 (1.2%) 4 (4.8%) 16 (19%) 38 (45.2%) 25 (29.8%)	3.6 (-) 3.4 (2-8.4) 20.5 (8.1-26) 18.6 (11.4-24) 21.8 (13-28)	**0.032**
Occupation Student Unemployed Employed Freelancer Labourer Pensioner	2 (2.4%) 31 (36.9%) 23 (27.4%) 15 (17.9%) 1 (1.2%) 12 (14.3%)	16 (14.5-17.5) 18.9 (5-25.7) 19.6 (15.2-23.8) 21.8 (19.7– 28.3) 26.1 (-) 5.8 (4.2-21.1)	0.071
Number of Partners 0 1 2-4 * >* 5	3 (3.6%) 47 (56%) 28 (33.3%) 6 (7.1%)	19 (12.1-19.4) 19.3 (4.8-25.2) 19.9 (14.6-24.2) 25.5 (20.6-27.7)	0.178

FSFI: Female Sexual Function Index; IQR: Inter Quartile Range; MASLD: Metabolic dysfunction-Associated Steatotic Liver Disease; kPa: kiloPascal; DM2: type 2 Diabetes Mellitus

*P* < 0.05 is considered statistically significant

Correlation between continuous variables and total FSFI score was assessed through the Spearman rho test and a statistically significant negative correlation was found between FSFI total score and age (*P* < 0.001, *P* = -0.368) or hypertension (*P =* 0.041, *P* = -0.224). Moreover, a negative correlation was found between MASLD and FSFI total score (*P =* 0.022, *P* = -0.249).

**Figure 1:**
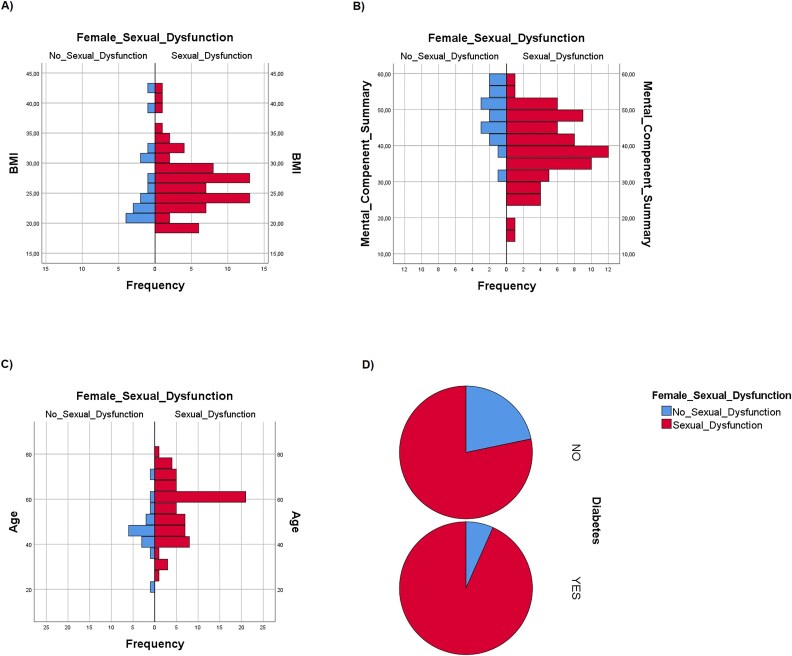
(A–D): Distribution of sexual dysfunctions (SD) and physiological sexual function (NO SD) in female patients according to body mass index (BMI) (A), mental component summary (MCS) (B), age (C) and type 2 diabetes mellitus (DM2) (D).

By means of logistic regression analysis we searched for independent positive or negative predictive factors of FSD and found that a high BMI and MCS were protective against SD (OR = 0.779, CI 95% = 0.640-0.948, *P =* 0.013 and OR = 0.840, CI 95% = 0.741-0.953, *P =* 0.007, respectively) (Figure 1A and B, respectively). On the other end, age (OR = 1.1146, CI 95% = 1.040–1.263, *P =* 0.006) and DM2 (OR = 120.894, CI 95% = 1.396–10 741, *P =* 0.035) were independent predictors of FSD (Figure 1C and D, respectively). Smoke, alcohol, PCS, adherence to MD and type of CLD did not seem to be predictive of SD (data not shown).

None of the patients declared any treatment for SD. Also, all of the 15 (18%) patients with DM2 were treated with diet alone. Of the 37 (44%) patients with hypertension, 30 were treated with angiotensin 2 receptor blockers and 7 with angiotensin converting enzyme (ACE) inhibitors. Data on dose and duration of treatment were not available.

### Sexual function in the male population

Median IIEF-ED score in our cohort of 123 CLD patients was 21 (15–26). By using the cut-off value of *<*25 to identify ED in men we found that 89/123 (72.3%) complained of ED. In particular, ED was mild in 52, moderate in 17 and severe in 20 patients. By analyzing the single domains of the IIEF-15 not related to the erectile function, we identified as likely areas of greatest sexual concern to a large number of participants, orgasm in 53/123 (43.1%), sexual desire in 86/123 (69.9%), satisfaction during the intercourse in 97/123 (78.9%), and general satisfaction in 63/123 (51.2%) patients (data not shown).

Table 4 shows IIEF-ED score differences between subgroups. In particular DM2, a lower disease duration, unemployment, and higher fibroscan values (ie, higher degree of liver fibrosis) were significantly associated to ED in our cohort of patients. Also, a significantly lower IIEF score was observed in MASLD than in nonMASLD patients.

**Table 4:** International Index of Erectile Function. Erectile Dysfunction (IIEF-ED) scores and clinical-demographic variables.

**Variables**	**N and Frequency (%)**	**IIEF-ED score (IQR)**	**p-value**
Smoke	60 (48.8%)	19.2 (15-26)	0.998
Alcohol	30 (24.4%)	19.8 (16-24.8)	0.839
CLD nonMASLD MASLD	62 (50.4%) 61 (49.6%)	23.5 (15.3-27) 20 (15-23)	**0.024**
Fibroscan kPa <7.2 k 7.3-12.5 12.5-17.5 >17.5	33 (26.8%) 27 (22%) 19 (15.4%) 44 (35.8%)	25 (19-27) 23 (20-27) 20 (14.5-23) 17 (12-22.3)	**0.003**
DM2	24 (19.5%)	13.3 (4.8-20.5)	**<0.001**
Hypertension	50 (40.7%)	18 (15-23)	0.086
Duration of Disease < 5 years 5-10 years * >* 10 years	67 (54.5%) 17 (13.8%) 39 (25.2%)	17.8 (15-23) 21.8 (18-27) 20.4 (16-27)	**0.044**
Education Unschooled Primary School Secondary School High School Degree	6 (4.9%) 8 (6.5%) 30 (24.4%) 50 (40.7%) 29 (23.6%)	14.8 (6-21.8) 9.6 (3.5-16.3) 20.1(16.3-25.8) 20.2 (17-26) 20.1 (16-26)	0.069
Occupation Student Unemployed Employed Freelancer Labourer Pensioner	2 (1.6%) 8 (6.5%) 34 (27.6%) 32 (26%) 19 (15.4%) 28 (22.8%)	16.5 (9.8-23.3) 10.4 (3.8-15.3) 22.3 (17.5-27) 19.7(16.8-25.3) 21.6 (19-26.5) 16 (8.8-22.3)	**0.006**
Number of Partners 0 1 2-4 * >* 5	8 (6.5%) 31 (25.2%) 45 (36.6%) 39 (31.7%)	17 (4.5-26.3) 18.4 (16-23) 18.4 (14-25) 21.2 (17-27)	0.336

IIEF-ED: International Index of Erectile Function-Erectile Dysfunction; IQR: Inter Quartile Range; CLD: Chronic Liver Disease; MASLD: Metabolic dysfunction-Associated Steatotic Liver Disease; kPa: kiloPascal; DM2: type 2 Diabetes Mellitus

*P* < 0.05 is considered statistically significant

A statistically significant negative correlation was found between IIEF-ED scores, and age (*P* < 0.001, *P* = -0.369), DM2 (*P* < 0.001, *P =* -0.329), or MASLD vs nonMASLD (*P* < 0.024, = -0.204), whereas a statistically significant positive correlation was found between IIEF-ED scores, and PCS (*P* < 0.001, *P =* 0.382), or MCS (*P =* 0.008, *P =* 0.236).

**Figure 2:**
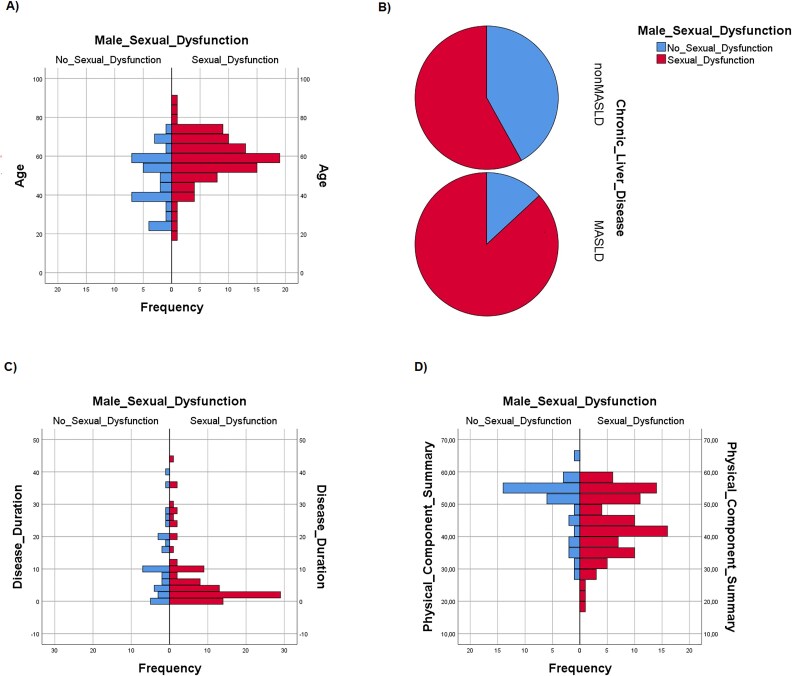
(A–D): Distribution of sexual dysfunctions (SD) and physiological sexual function (NO SD) in male patients according to age (A), type of chronic liver disease (CLD) (B), disease duration (C) and physical component summary (PCS) (D).

By means of logistic regression analysis we searched for independent positive or negative predictive factors of SD and found that age (OR = 1088, CI 95% = 1032-1.148, *P =* 0.002) and MASLD aetiology (OR = 4.075, CI 95% = 1.120-14.828, *P =* 0.033) were independent risk factors for SD in males (Figure 2A and B, respectively). On the other hand, disease duration (OR = 0.393, CI 95% = 0.187-0.822. *P =* 0.013) and PCS (OR = 0,928, CI 95% = 0,865-0,995, *P =* 0.036) were independent protective factors against SD (Figure 2C and D, respectively). Smoke, alcohol, hypertension BMI, DM2, MCS and adherence to MD were not independent predictors of SD (data not shown).

None of the patients declared any treatment for SD. Twenty-four (19.5%) patients had DM2 which was controlled with diet in 18 patients and with insulin in six, whereas 50 (40.7%) patients had hypertension which was treated with angiotensin 2 receptor blockers in 42 and with ACE inhibitors in 8. We do not have information on dose and duration of treatment.

## Discussion

In this study we first sought to evaluate the prevalence of SD in patients with CLD and found that both females and males suffering from CLD had a high prevalence of SD as assessed by the FSFI-19 and IIEF-15 questionnaires. Specifically, in the female population, 68/84 (80.9%) were at risk for FSD with an FSFI total score < 26.5. In detail, the areas of greatest sexual concern to the majority of patients were sexual desire (94%), arousal (82.1%), lubrication (73.8%), orgasm (79.7%), intercourse satisfaction (69%), and dyspareunia (80.9%). On the other hand, 89/123 males (72.3%) reported ED which was mild in 52, moderate in 17 and severe in 20 patients. Moreover, by specifically analyzing the domains of the IIEF-15 not related to the erectile function, we highlighted that the areas of greatest sexual concern to the majority of participants where orgasm (43.1%), sexual desire (69.9%), satisfaction during intercourse (78,9%), and general satisfaction (51.2%).

The prevalence of SD found in this study is higher than that reported in the general population which is close to 40%–50% for females, independently of age, and 1% to 15% in the age-matched male population.^1^ Moreover, our results are in agreement with previous reports showing that patients with CLD are at higher risk of developing SD^16–25^ with a prevalence of ED ranging from 24% to 85%.^23^ In particular, a report by Lyn et al, using cross-sectional data from the National Health and Nutrition Examination Survey conducted between 2001 and 2004 to examine the health of those over 20 years of age, found that MASLD was significantly associated to a significant risk of ED with a prevalence of 29% in those people with a high United States Fatty Liver Index.^36^ Also, Tarik et al described a prevalence of ED ranging from 40% to 70 % in patients with MASLD^34^ which is comparable to the prevalence of 60% found in our similar group of patients. Similarly, Lee et al reported a prevalence of SD in almost 68% of women suffering from MASLD which is similar to the prevalence of 59% observed in our cohort.^35^

In our study, prevalence of SD was significantly higher in patients with MASLD than in those with nonMASLD aetiology (ie, 89% vs 64%, *P* < 0.001). These figures are similar to those reported in other studies^35^^,^^37^ evaluating SD in patients with CLD, even though, to our knowledge, there are no studies directly comparing MASLD with nonMASLD as to the prevalence of SD. Interestingly, at the logistic regression analysis, in the male population, but not in females, MASLD aetiology was an independent risk factor for SD with an OR = 4.075, CI 95% = 1.120–14.828. This reinforces the concept that patients with MASLD are at risk for SD. In fact, in a study by Duman et al in 40 patients with NAFLD, the OR for SD was of 2.4 (95% CI 1.08–5.5).^37^ Also, Eren and Horsanaly in their study in over 350 patients reported that NAFLD significantly correlated with low IIEF-5 scores.^38^ Interestingly, MASLD has also been associated with Peyronie’s disease, probably due to MASLD-induced alteration of local penile microenvironment, with chronic, low-grade inflammation leading to endothelial dysfunction, increased vascular permeability, generation of reactive oxygen species and fibrosis.^39^

In our female population, hypertension was significantly associated to lower FSFI scores. That hypertension is a risk factor for SD in women has been reported in a number of studies.^40^^,^^41^ High blood pressure may cause sexual dysfunction in women by lowering the blood flow to the vagina and lowering nitric oxide (NO) levels thus affecting smooth muscle relaxation. This might lead to decreased sex drive or arousal, vaginal dryness and difficulty in achieving an orgasm.^41^ However, the relationship between hypertension and FSD has never been investigated in the context of CLD and, therefore, our finding of a significant association between hypertension and SD in females in this clinical setting should be confirmed in larger longitudinal studies.

Quality of life as well as interpersonal relationships are severely and negatively influenced by SD both in males and females. We found that MCS in women and PCS in men were independent determinants of a normal sexual function. Our data further strengthen the concept that physical well-being is strongly related to a satisfying sexual activity.^42^ In fact, evidence suggests that sexual health should be used as a surrogate marker for systemic health thus facilitating prevention, diagnosis and treatment of a number of chronic diseases.^3^ Moreover, sexual function in females is well-known to strictly depend on emotional and psychological factors^42^ and this concept is indeed included in the definition of FSD which is described as a multidimensional disorder in which physiological, clinical, psychological and socio-cultural factors play a pathogenic role.^4^

Because MD has been demonstrated to be exert beneficial effects on sexual function in both males and females, specifically in people with metabolic disorders,^6–10^ we also evaluated the role of adherence to MD in our cohort of CLD patients. The adherence to MD was low with a median score of 4 out of a total of 9. MASLD-patients had a significantly lower adherence score to MD compared with nonMASLD-patients [(3.0 (2–4) vs 4.0 (3–5, *P* < 0.004). However, as assessed by means of multiple logistic regression analysis, adherence to MD did not emerge as a factor independently predictive of sexual health both in males and females. The lack of any other study describing the effects of Mediterranean dietary pattern on CLD does not allow to make comparison. Therefore, before drawing any conclusion on the role of MD in the development of SD in CLD patients, there is the need of longitudinal studies specifically designed to address this issue.

Whether BMI is a significant risk factor for female SD is controversial.^43–46^ In our study we found that higher BMI category in the female population was a significant predictor of a better sexual health (ie, higher FSFI total score). A recent study by Faubion et al demonstrated a significant association between high BMI category and decreased sexual health as assessed by FSFI, even though BMI was not an independent risk factor for SD by multiple logistic regression analysis.^47^ However, similarly to our finding, a study investigating associations between sexual activity and weight status found that overweight women had more frequent sexual activity compared to their normal weight counterparts.^48^ The Authors suggested that a possible explanation for this might be that people in stable relationships tend to gain weight over time.

The mechanisms whereby CLD may cause SD are several. The degree of liver function impairment is a main determinant of SD in CLD patients.^11^ In support of this concept, Sorrel and Brown demonstrated that in patients with decompensated liver cirrhosis, before, receiving OLT, SD was independent of the etiology of the underlying CLD but rather due to the advanced stage of liver cirrhosis.^49^ Also, a substantial improvement of sexual performance has been demonstrated in patients with end stage liver disease following OLT.^19^^,^^20^ Altered hypotalamus-pituitary-gonadal axis, changes in the estrogen-over-androgen ratio and increased serum levels of sex hormone binding globulin together with disturbed mental and psychological conditions may all play relevant pathogenic role in this clinical setting.^21^ However, also the etiology of CLD may play a role. Altered inter-relational aspects due to the worry of transmitting an infectious agent together with oxidative stress, chronic inflammation, or cryoglobulinemia-related endothelial dysfunction may be a cause of SD in HBV- or HCV- related CLD.^24^^,^^50^^,^^51^ Also, as far as MASLD is concerned, endothelial dysfunction, which plays a relevant pathogenic role in ED,^52^ has been shown as one of the earliest events associated with liver fat accumulation and subsequent liver damage.^53^ Moreover, MASLD patients have a marked endothelial NO synthase dysfunction^54^ which might be accounted for by an altered insulin signalling caused by insulin resistance, a common finding in MASLD patients.^55^ Penile erection and its maintenance are strongly dependent on endothelium-produced NO, and as NO availability decreases, ED becomes impaired.^56^ Finally, MASLD might be triggered by environmental exposure to endocrine disrupting chemicals such as bisphenol A,^57^ which has been shown to affect sexual function by decreasing serum levels of a number of sexual hormones.^58^

The major limitation of this study is that, due to its cross-sectional nature, no conclusions can be drawn about cause and effect because, as with most epidemiological studies, residual bias due to uncontrolled covariates are a possibility. However, the major strengths of this study include the use of a validated instrument to assess sexual dysfunction, the comprehensive assessment of numerous aspects of life related to sexual function (QoL, and lifestyle habits including dietary patterns). Also, to our knowledge this is the only study evaluating SD in a large cohort of patients with well-compensated CLD of both sexes, comparing MASLD vs nonMASLD subjects.

In conclusion, patients with well compensated CLD, both females and males, have a high prevalence of SD with a difference in the clinical predictors between sexes. In particular, in females, age and DM2 were significant predictors of FSD risk whereas BMI and MCS were protective. In males, age and MASLD aetiology were significant predictors of SD whereas PCS and disease duration were protective against SD. Based on the results of this study we suggest that the assessment of sexual function in CLD patients through the use of validated questionnaires might be useful to reach an early diagnosis and start a timely treatment.

## References

[1] McCabe MP, Sharlip ID, Lewis R, et al. Incidence and prevalence of sexual dysfunction in women and men: a consensus statement from the fourth international consultation on sexual medicine 2015. J Sex Med. 2016;13(2):144–152. 10.1016/j.jsxm.2015.12.034 26953829

[2] Laumann EO, Nicolosi A, Glasser DB, et al. Sexual problems among women and men aged 40-80 y: prevalence and correlates identified in the global study of sexual attitudes and Behaviors. Int J Impot Res. 2005;17(1):39–57. 10.1038/sj.ijir.3901250 15215881

[3] Jannini EA . SM = SM: the Interface of systems medicine and sexual medicine for facing non-communicable diseases in a gender-dependent manner. Sex Med Rev. 2017;5(3):349–364. 10.1016/j.sxmr.2017.04.002 28596070

[4] McCabe MP, Sharlip ID, Atalla E, et al. Definitions of sexual dysfunctions in women and men: a consensus statement from the fourth international consultation on sexual medicine 2015. J Sex Med. 2016;13(2):135–143. 10.1016/j.jsxm.2015.12.019 26953828

[5] Corona G, Cucinotta D, Di Lorenzo G, et al. The Italian Society of Andrology and Sexual Medicine (SIAMS), along with ten other Italian scientific societies, guidelines on the diagnosis and management of erectile dysfunction. J Endocrinol Investig. 2023;46(6):1241–1274. 10.1007/s40618-023-02015-5 36698034 PMC9876440

[6] Mollaioli D, Ciocca G, Limoncin E, et al. Lifestyles and sexuality in men and women: the gender perspective in sexual medicine. Reprod Biol Endocrinol. 2020;18(1):10. 10.1186/s12958-019-0557-9 32066450 PMC7025405

[7] Maiorino MI, Bellastella G, Giugliano D, Esposito K. From inflammation to sexual dysfunctions: a journey through diabetes, obesity, and metabolic syndrome. J Endocrinol Investig. 2018;41(11):1249–1258. 10.1007/s40618-018-0872-6 29549630

[8] Maiorino MI, Bellastella G, Chiodini P, et al. Primary prevention of sexual dysfunction with Mediterranean diet in type 2 diabetes: the MÈDITA randomized trial. Diabetes Care. 2016;39(9):e143–e144. 10.2337/dc16-0910 27352954

[9] Bauer SR, Breyer BN, Stampfer MJ, Rimm EB, Giovannucci EL, Kenfield SA. Association of diet with erectile dysfunction among men in the health professionals follow-up study. JAMA Netw Open. 2020;3(11):e2021701. 10.1001/jamanetworkopen.2020.21701 33185675 PMC7666422

[10] Towe M, La J, El-Khatib F, Roberts N, Yafi FA, Rubin R. Diet and female sexual health. Sex Med Rev. 2020;8(2):256–264. 10.1016/j.sxmr.2019.08.004 31669123

[11] Romano L, Granata L, Fusco F, et al. Sexual dysfunction in patients with chronic gastrointestinal and liver diseases: a neglected issue. Sex Med Rev. 2022;10(4):620–631. 10.1016/j.sxmr.2021.02.002 34353738

[12] Romano L, Pellegrino R, Sciorio C, et al. Erectile and sexual dysfunction in male and female patients with celiac disease: a cross-sectional observational study. Andrology. 2022;10(5):910–918. 10.1111/andr.13186 35419983 PMC9324123

[13] Romano L, Fonticelli M, Miranda A, et al. Sexual dysfunctions in inflammatory bowel disease: role of mediterranean diet and quality of life. Andrology 2024 Nov 3:1–9. 10.1111/andr.13791. Online ahead of print. PMC12476212 39492590

[14] Noureddin M, Wei L, Castera L, Tsochatzis EA. Embracing change: from nonalcoholic fatty liver disease to metabolic dysfunction-associated Steatotic liver disease under the steatotic liver disease umbrella. Clin Gastroenterol Hepatol. 2024;22(1):9–11. 10.1016/j.cgh.2023.09.034 37848118

[15] Caussy C, Aubin A, Loomba R. The relationship between type 2 diabetes, NAFLD, and cardiovascular risk. Curr Diab Rep. 2021;21(5):15. 10.1007/s11892-021-01383-7 33742318 PMC8805985

[16] Hawksworth DJ, Burnett AL. Nonalcoholic fatty liver disease, male sexual dysfunction and infertility: common links, common problems. Sex Med Rev. 2020;8(2):274–285. 10.1016/j.sxmr.2019.01.002 30898592

[17] Paternostro R, Heinisch BB, Reiberger T, et al. Erectile dysfunction in cirrhosis is impacted by liver dysfunction, portal hypertension, diabetes and arterial hypertension. Liver Int. 2018;38(8):1427–1436. 10.1111/liv.13704 29368385 PMC6766949

[18] Philonenko S, Rivière P, Mallet M, et al. Neurocognitive impairment is associated with erectile dysfunction in cirrhotic patients. Dig Liver Dis. 2019;51(6):850–855. 10.1016/j.dld.2019.03.030 31031175

[19] Chien YC, Chiang HC, Lin PY, Chen YI. Erectile function in men with end-stage liver disease improves after living donor liver transplantation. BMC Urol. 2015;15(1):83. 10.1186/s12894-015-0078-6 26268947 PMC4535392

[20] Chiang HC, Chien YC, Lin PY, Lee HL, Chen YL. Assessing men with erectile dysfunction before and after living donor liver transplantation in real-world practice: integrating laboratories into clinical setting. PLOSone. 2018;13(11):1–11, e0206438. 10.1371/journal.pone.0206438 PMC6245674 30458009

[21] Neong SF, Billington E, Congly SE. Sexual dysfunction and sex hormone abnormalities in patients with cirrhosis: review of pathogenesis and management. Hepatology. 2019;69(6):2683–2695. 10.1002/hep.30359 30468515

[22] Zang G, Sun X, Sun Y, et al. Chronic liver diseases and erectile dysfunction. Front Public Health. 2023;6(10):1–12, 1092353. 10.3389/fpubh.2022.1092353 PMC9853559 36684968

[23] Karaivazoglou K, Tsermpini E-E, Assimakopoulos K, Triantos C. Sexual functioning in patients with chronic hepatitis C: a systematic review. Eur J Gastroenterol Hepatol. 2017;29(11):1197–1205. 28834789 10.1097/MEG.0000000000000949

[24] Liao X, Zhao S, Yin J, et al. Sexual dysfunction in patients with chronic hepatitis B: prevalence and risk factors. J Sex Med. 2022;19(2):207–215. 10.1016/j.jsxm.2021.11.016 34969615

[25] Di Stasi V, Maseroli E, Rastrelli G, et al. SHBG as a marker of NAFLD and metabolic impairments in women referred for oligomenorrhea and/or hirsutism and in women with sexual dysfunction. Fron Endocrinol. 2021 Mar 29;12:1–11, 641446. 10.3389/fendo.2021.641446 PMC8040974 33854482

[26] Dayi T, Ozgoren M. Effects of the Mediterranean diet on the components of metabolic syndrome. J Prev Med Hyg. 2022;63(2 Suppl 3):E56–E64. 10.15167/2421-4248/jpmh2022.63.2S3.2747 36479500 PMC9710414

[27] Child CG, Turcotte JG. Surgery and portal hypertension. Major Probl Clin Surg. 1964;1:1–85. 4950264

[28] Pugh RN, Murray-Lyon IM, Dawson JL, Pietroni MC, Williams R. Transection of the oesophagus for bleeding oesophageal varices. Br J Surg. 1973;60(8):646–649. 10.1002/bjs.1800600817 4541913

[29] Foucher J, Chanteloup E, Vergniol J, et al. Diagnosis of cirrhosis by transient elastography (FibroScan): a prospective study. Gut. 2006;55(3):403–408. 10.1136/gut.2005.069153 16020491 PMC1856085

[30] Wiegel M, Meston C, Rosen R. The female sexual function index (FSFI): cross.Validation and development of clinical cutoff scores. J Sex Marital Ther. 2005;31(1):1–20. 10.1080/00926230590475206 15841702

[31] Rosen RC, Riley A, Wagner G, Osterloh IH, Kirkpatrick J, Mishra A. The international index of erectile function (IIEF): a multidimensional scale for assessment of erectile dysfunction. Urology. 1997;49(6):822–830. 10.1016/S0090-4295(97)00238-0 9187685

[32] Trichopoulou A, Costacou T, Bamia C, Trichopoulos D. Adherence to a Mediterranean diet and survival in a Greek population. N Engl J Med. 2003;348(26):2599–2608. 10.1056/NEJMoa025039 12826634

[33] Ware J Jr, Kosinski M, Keller SD. A 12-item short-form health survey: construction of scales and preliminary tests of reliability and validity. Med Care. 1996;34(3):220–233. 10.1097/00005650-199603000-00003 8628042

[34] Kani HT, Sener TE, Aykut UE, et al. Causes of erectile dysfunction in non-alcoholic fatty liver disease. Hepatol Forum. 2021;2(2):60–63. 35783901 10.14744/hf.2021.2021.0012 PMC9138920

[35] Lee JY, Shin DW, Oh JW, et al. Non-alcoholic fatty liver disease as a risk factor for female sexual dysfunction in premenopausal women. PLoS One. 2017;12(8):E0182708. 10.1371/Journal.Pone.0182708 28854246 PMC5576732

[36] Lyn Y, Wu X, Chen Z. Association between nonalcoholic fatty liver disease and erectile dysfunction among American adults from the National Health and nutrition examination survey: a cross-sectional study. Int J Impotence Res. 2024 May 23:1–9. 10.1038/s41443-024-00914-6 38783042

[37] Duman DG, Bicakci E, Celikel CA, Akbal C. Nonalcoholic fatty liver disease is associated with erectile dysfunction: a prospective pilot study. J Sex Med. 2016;13(3):383–388. 10.1016/j.jsxm.2015.12.030 26853046

[38] Eren H, Horsanali MO. The independent association of nonalcoholic fatty liver disease with lower urinary tract symptoms/benign prostatic hyperplasia and erectile function scores. BJU Intern. 2019;124(2):329–335. 10.1111/bju.14753 30900792

[39] Crocetto F, Barone B, Manfredi C, et al. Are insulin resistance and NAFLD associated with Peyronie’s disease? A pilot study J Physiol Pharmacol. 2022;73(1):15–24. 10.26402/jpp.2022.1.05 35639037

[40] Dilixiati D, Cao R, Mao Y, et al. Association between cardiovascular disease and risk of female sexual dysfunction: a systematic review and meta-analysis. Eur J Prev Cardiol. 2024;31(7):702–800. 10.1093/eurjpc/zwae042 38297501

[41] Lou IX, Chen J, Ali K, Chen Q. Relationship between hypertension, antihypertensive drugs and sexual dysfunction in men and women: a literature review. Vasc Health Risk Manag. 2023 Nov 3;19:691–705. 10.2147/VHRM.S439334 37941540 PMC10629452

[42] Lindau ST, Schumm LP, Laumann EO, Levinson W, O'Muircheartaigh CA, Waite LJ. A study of sexuality and health among older adults in the United States. N Engl J Med. 2007;357(8):762–774. 10.1056/NEJMoa067423 17715410 PMC2426743

[43] Esposito K, Ciotola M, Giugliano F, et al. Association of body weight with sexual function in women. Int J Impot Res. 2007;19(4):353–357. 17287832 10.1038/sj.ijir.3901548

[44] Mozafari M, Khajavikhan J, Jaafarpour M, Khani A, Direkvand-Moghadam A, Najafi F. Association of body weight and female sexual dysfunction: a case control study. Iran Red Crescent Med J. 2015;17(1):e24685. 10.5812/ircmj.24685 25763278 PMC4341402

[45] Bajos N, Wellings K, Laborde C, Moreau C, Group CSF. Sexuality and obesity, a gender perspective: results from French national random probability survey of sexual behaviours. BMJ. 2010;340(jun15 1):1–9, c2573. 10.1136/bmj.c2573 20551118 PMC2886194

[46] Kadioglu P, Yetkin DO, Sanli O, Yalin AS, Onem K, Kadioglu A. Obesity might not be a risk factor for female sexual dysfunction. BJU Int. 2010;106(9):1357–1361. 10.1111/j.1464-410X.2010.09348.x 20394615

[47] Faubion SS, Fairbanks S, Cl K, et al. Association between body mass index and female sexual dysfunction: a cross-sectional study from the data registry on experiences of aging, menopause and sexuality. J Sex Med. 2020;17(10):1971–1980. 10.1016/j.jsxm.2020.07.004 32771351 PMC7662836

[48] Smith L, Yang L, Forwood S, et al. Associations between sexual activity and weight status: findings from the English longitudinal study of ageing. PLoS One. 2019;14(9):1–11, e0221979. 10.1371/journal.pone.0221979 31498846 PMC6733459

[49] Sorrel JH, Brown JR. Sexual functioning in patientswith end-stage liver diseases before and after transplantation. Liver Transpl. 2006;12(10):1473–1477. 10.1002/lt.20812 16741902

[50] Elshimi E, Morad W, Mohamad NE, Shebl N, Waked I. Female sexual dysfunction among Egyptian patients with chronic hepatitis C. J Sex Med. 2014;11(3):768–775. 10.1111/jsm.12412 24283464

[51] Elshimi E, Morad W, Mohamad NE. Male sexual dysfunction among Egyptian patients with chronic Hepatitis C Virus infection before and after direct-acting antiviral drugs. J Sex Med. 2019;16(3):402–409. 10.1016/j.jsxm.2019.01.309 30846113

[52] Bivalacqua TJ, Usta MF, Champion HC, Kadowitz PJ, Hellstrom WJG. Endothelial dysfunction in erectile dysfunction: role of the endothelium in erectile physiology and disease. J Androl. 2003;24(6 Suppl):S17–S37. 10.1002/j.1939-4640.2003.tb02743.x 14581492

[53] Pasarin M, La Mura V, Gracia-Sancho J, et al. Sinusoidal endothelial dysfunction precedes inflammation and fibrosis ina model of NAFLD. PLoS One. 2012;7(4):e32785. 10.1371/journal.pone.0032785. 22509248 PMC3317918

[54] Persico M, Masarone M, Damato A, et al. Non alcoholic fatty liver disease and eNOS dysfunction in humans. BMC Gastroenterol. 2017;17(1):35. 10.1186/s12876-017-0592-y 28264657 PMC5340006

[55] Kim JA, Montagnani M, Koh KK, et al. Reciprocal relationships between insulin resistance and endothelial dysfunction: molecular and pathophysiological mechanisms. Circulation. 2006;113(15):1888–1904. 16618833 10.1161/CIRCULATIONAHA.105.563213

[56] Burnett AL, Lowenstein CJ, Bredt DS, Chang TS, Snyder SH. Nitric oxide: a physiologic mediator of penile erection. Science. 1992;257(5068):401–403. 1378650 10.1126/science.1378650

[57] Federico A, Dallio M, Gravina AG, et al. The bisphenol a induced oxidative stress in nonalcoholic fatty liver disease in male patients: a clinical strategy to antagonize the progression of the disease. Int J Environ Res Public Health. 2020;17(10):3369. 10.3390/ijerph17103369 32408667 PMC7277712

[58] Li D, Zhou Z, Qing D, et al. Occupational exposure to bisphenol a (BPA) and the risk of self-reported male sexual dysfunction. Hum Reprod. 2010;25(2):519–527. 10.1093/humrep/dep381 19906654

